# Preparing for the unexpected: a comparative study of policies addressing post-terror health reactions in Norway and France

**DOI:** 10.1186/s13033-023-00582-x

**Published:** 2023-05-26

**Authors:** Lisa Govasli Nilsen, Lise Eilin Stene

**Affiliations:** 1 Norwegian Center for Violence and Traumatic Stress Studies, Oslo, Norway; 2 Department of Sociology and Political Science, Norwegian University of Science and Technology, NTNU, Trondheim, Norway

**Keywords:** Terrorism, Disasters, Healthcare systems, Health contingency

## Abstract

**Background:**

In the wake of terrorist attacks, protecting the health and psychosocial wellbeing of those affected and the general population, are important tasks for the healthcare system. The responses to such emergencies are often complex, including different phases and many actors, and may unveil insufficiencies that incite reforms to existing systems. Recently, initiatives have been promoted to strengthen cooperation and coordination regarding the governance of health threats in Europe. Comparative research is requested on how states prepare for health emergencies such as terrorist attacks. This study investigated how governments in two European countries with universal health coverage prepared to address the civilian population’s health needs after terrorist attacks, and the factors that contributed to shaping their chosen approach.

**Methods:**

Utilizing document analysis and Walt and Gilson’s model for the analysis of health policy, national plans for post-terror health responses in Norway and France were studied with a focus on context, process, content, and actors.

**Results:**

Whereas target groups for psychosocial care and certain measures were similar in both cases, the contents of prescribed policies and the actors responsible for enacting them differed. One of the most distinct differences was to what extent specialized mental healthcare was relied upon to provide psychosocial follow-up in the emergency phase. In the French approach, specialized mental healthcare practitioners, such as psychiatrists, psychologists and psychiatric nurses, provided early psychosocial support. In contrast, the Norwegian approach relied on interdisciplinary primary care crisis teams in the local municipalities to provide early psychosocial support, with further involvement of specialized mental healthcare if this was considered necessary. Historical, political, and systemic differences contributed to the variation in the countries’ responses.

**Conclusions:**

This comparative study highlights the complexity and diversity of health policy responses to terrorist attacks across countries. Moreover, challenges and opportunities for research and health management in response to such disasters, including possibilities and potential pitfalls for the coordination of this work across Europe. An important first step could be to map out existing services and practices across countries to better understand if and how common core elements for psychosocial follow-up might be implemented internationally.

**Supplementary Information:**

The online version contains supplementary material available at 10.1186/s13033-023-00582-x.

## Background

In recent decades, Europe has experienced several terrorist attacks which left healthcare needs in their wakes. Terrorist attacks in generally peaceful democracies are typically followed by policy initiatives designed to meet the needs of those directly affected and the general population. Alongside initiatives established before a disaster, new measures, or reforms of existing systems, are often initiated to better meet a population’s needs [1, 2]. Given that terrorism is often transnational in character, there has also been a push for coordinating policies in different countries [3, 4], and initiatives have been promoted to enable stronger cooperation regarding the governance of health threats in Europe specifically [5]. Yet, comparative research is needed to understand how different countries plan for their health contingency in events of terrorism. This is important, both in order to inform discussions of best practices for disaster follow-up, and to expose possibilities and potential pitfalls for increased transnational cooperation.

### Health policy in the wake of political violence

Following the 9/11 attacks in the United States, scholars and policymakers have devoted increased attention to emergency preparedness [4, 6–9]. This includes discussions on how states can best prepare to meet the needs of those directly affected by such events [10], and policies have been developed to this end. In this context, policy is a plan that “…sets priorities and guides resource allocation” [11, p. 622]. The process of policy creation can be understood as cyclical, typically involving agenda setting, policy formation, policy implementation, and policy review [12]. Furthermore, there is an important temporal aspect, as policies are intended to resolve a specific issue within a certain time [12]. Policy plans are normative to the extent that they prescribe policy makers’ and stakeholders’ intentions.

Policies aimed at meeting needs in a post-terror context are part of a state’s emergency preparedness. Public health emergency preparedness could be understood as: “..the capacity of the public health and healthcare systems, communities, and individuals to prevent, protect against, quickly respond to, and recover from health emergencies, particularly those whose scale, timing, or unpredictability threatens to overwhelm routine capabilities” [13, p. 9]. However, what is required to meet the needs of a post-disaster situation remains unclear [14]. There is debate surrounding emergency preparedness in the public health sector, the specific problems it should solve, the available solutions, and the timing with which they should be implemented [13, 14].

Terrorist attacks are man-made disasters with political aims [15], which could have implications for the problems that need solving. The scale of an attack and other characteristics may affect potential challenges and solutions, and responding to large scale terrorist attacks is often a complex task involving several actors. In addressing terrorist attacks and similar disasters, there is a strong focus on the need for psychosocial follow-up [4, 7], but also on other types of medical care, protection, and recognition [16]. There are international guidelines addressing healthcare provision following terrorism and other disasters [17], including psychosocial care [18] and recommendations for the field application of the Psychological First Aid method [19]. Yet, little is known about the extent to which such guidelines are followed and how they are implemented. There is research suggesting that there are differences in how countries in Europe, including the ones investigated in this study, have provided psychosocial follow-up after terrorism [20]. More in-depth research is needed, however, into the planning process of such endeavors, to understand how different countries plan beforehand to meet their population’s needs following terrorism, and why they plan in particular ways.

In attempting to understand why countries plan the way they do, it is central to note that responses following an attack provide an opportunity to learn, both in the country affected and elsewhere [4]. In this sense, such events may alter how the political system subsequently handles similar incidents. To understand the development of policies in this area, it could therefore be relevant to take Kingdon’s [2] conceptualization of political agenda setting into account. Kingdon [2] asserts that political agenda setting constitutes three types of processes: problems, policies, and politics. The “problem stream” describes how issues are brought to the fore when they become problems for the system, such as a crisis. The “policy stream” refers to how accumulation of knowledge over time push issues onto the agenda. Finally, the “politics stream” refers to how alterations in public attitudes and changes in the political systems may push certain issues on the agenda.

### Aims and objectives

The aim of this study was to investigate how governments in generally peaceful democracies prepare to address the population’s health needs after a terrorist attack, and the factors that contribute to shaping their chosen approach. The objective was to examine plans addressing a population’s post-terror health and wellbeing. Applying an explorative approach, the study responded to the following research questions:

1. How are health-related needs in the civilian population understood and addressed in national policy plans for disaster follow-up generally, and in post-terror responses more specifically, in Norway and France?
2. How can differences in the two countries’ approaches be understood?

## Methods

### Case selection

This study compared policies and plans addressing health responses related to four terrorist attacks in Norway and France. Both nations are stable and relatively peaceful democracies with universal health coverage. Terrorist attacks in these countries may differ from attacks in unstable or conflict settings in terms of the state’s capacity to respond [21]. Given that the events occurred in stable democracies, one should expect state capacity to respond to the attacks to be high. Nevertheless, all democracies will not necessarily perform in similar ways post-disaster, and comparing the approach in two countries which have experienced some of the largest terrorist attacks in Western Europe in recent decades, is useful for understanding more about different approaches to health contingency.

#### The terrorist attacks

Norway and France have experienced some of the larger terrorist attacks in Western Europe in recent decades [22]. The attacks being studied affected victims of all ages, with those on Utøya island and in Nice affecting many children and youths. Furthermore, all attacks were met by broad responses to health concerns in victims and others, albeit within different healthcare systems [20]. An overview of the characteristics of the attacks can be found in Table 1.

**Table 1:** Overview of the terrorist attacks

Place and date of attack	Perpetrators	Mode of attack	Directly affected
Oslo and Utøya island, Norway on July 22, 2011	One terrorist with sympathies to the extreme right and a stated aim of targeting the Labor Party in an attempt to protect Norway against what he perceived to be liberal policies towards Islam He was arrested and sentenced to preventive detention for acts of terror, premediated murder, and attempted murder	• Bomb attack at the governmental quarters • Shooting spree at the Norwegian Labor Youth summer camp	• Government officials • Passers-by at governmental quarters • Youth camp participants 77 people were killed, 69 of them at the summer camp
Paris, France on January 7–9, 2015	A group of attackers. The attackers were shot and killed by the police, and the Al Qaeda branch in the Arabian Peninsula later claimed responsibility	• Shooting spree at satirical newspaper Charlie Hebdo • Attack and hostage taking at a kosher supermarket • One other hostage situation • Two other shooting incidents	• Satirical journalists • Kosher supermarket customers • Police officers • Passers-by 18 people were killed, including 12 at the newspaper and 4 at the kosher supermarket
Paris, France on November 13, 2015	A group of attackers. The attackers were killed during the police operation, and responsibility for the attacks was later claimed by the Islamic State militant group	• Suicide bombing in area around a football stadium • Shootings in four different locations, predominately bars and restaurants, in central Paris • Suicide bombing at a restaurant in central Paris • Shooting/attempted suicide attack at the concert venue Bataclan	• Spectators and passers-by at the football stadium • Guests at bars/restaurants and passers-by in central Paris • Concert-goers at Bataclan 130 people were killed
Nice, France on July 14, 2016	One attacker who was killed by the police. His links to terrorist organizations are disputed. The Islamic State (IS) claimed responsibility for the attack	• Lorry attack	• Crowd watching fireworks in celebration of the French National day 86 people were killed

#### History of terrorism

The two countries differ in their previous history of terrorism. The Norwegian attack represented a singular event without precedent in the country’s recent history, whereas the French attacks represent three sets of events in a longer history of recurring terrorist attacks [22].

#### The political systems

Both countries are unitary states. Norway has three relevant levels of government. At the national level are government ministries, as well as directorates under their auspices. At the sub-national level are the counties. In 2011, there were 19 counties in Norway. At the local level, the municipalities are the key units. There were 430 municipalities in Norway in 2011, with populations ranging from 220 to 612,314 [23]. As is common in Nordic countries [24], the principle of local self-government is central in Norway. Whereas primary care, such as general practitioners (GPs), is provided through the municipalities, specialized care is organized through four regional health authorities. In France, the important levels of government, are similar at the national level, with the ministries and directorates, the sub-national level, most importantly the Security and Defense Zones, and locally, in the departments. There were seven Security and Defense Zones in metropolitan France, as well as five overseas, in 2016, and 101 departments, the latter with populations ranging from 76,422 to 2,603,723 [25]. In addition, the Regional Health Authorities are central in the provision of post-disaster healthcare. The French have a fused system, in which representatives from the central government, known as prefects, are placed in each department to supervise the local governments [24].

#### Healthcare system

Both countries have universal healthcare. In Norway, this is organized as tax-financed healthcare, with a high degree of public financing, whereas the healthcare system in France is based on a social health insurance model covering nearly the entire population [26, 27]. Both countries spend a high share of GDP on healthcare—in 2018 this amounted to 10.1% for Norway and 11.3% for France [28]. An important difference between the two healthcare systems is their sheer size in terms of population covered. The healthcare system in Norway is a semi-decentralized system providing services to a population of approximately 5 million in one the least densely populated countries in Europe. The healthcare system in France, provides services to a population of approximately 66 million in metropolitan France, as well as territories overseas. The French system is more centralized, as compared to Norway, but with increasing responsibility placed on sub-national levels. Regarding access to healthcare, both countries have geographic and social differences [26, 27, 29].

### Research design

Our data included national plans and guidelines from the French and Norwegian authorities guiding the response to healthcare needs in the civilian population post-terror. Documents that were either valid at the time of the attacks, or that described measures initiated shortly thereafter, were included in the analysis. Relevant documents were collected through the following steps:

1. Review of academic and grey literature on post-terror response in France and Norway to identify relevant plans and actors.
2. Review of the webpages of the Norwegian Ministry of Health and Care Services [30], the Norwegian Directorate of Health [31], the French Ministry of Solidarity and Health [32], and the French Government [33] to identify plans focused on terrorism follow-up. Webpages were surveyed in English, and in the respective languages of each country.
3. Personal communication with stakeholders from the authorities, who provided quality control for the relevance of documents already collected and additional documents where necessary.

For documents published after the attacks, individual assessments were made regarding their relevance, since the period after a crisis can often be characterized by policy changes. Given the aim of this study, it was relevant to include some documents published post-disaster. Concurrently, the objective of the study was to study planning, not implementation. Evaluations were therefore not included. Only policy documents addressing health responses to terrorist attacks and/or similar disasters were analyzed. Additionally, only documents that were publicly available could be analyzed. In the case of terror response there may also be classified documents, or documents no longer publicly available due to updates and replacement.

The Norwegian data were analyzed in their original form, as Norwegian is the authors’ mother tongue. The French documents were subject to review and selection by the second author, who is fluent in French, before being translated into English by an external translator prior to the full analysis.

### Analysis

All documents were analyzed by document analysis, using a combination of content analysis and thematic analysis [34], as recommended by Bowen [35]. When conducting cross-country comparison of policies, typologies can be useful to organize the nearly endless amounts of information [36]. Walt and Gilson [37] suggest a model for the analysis of health policy, focusing on the interrelated concepts of context, process, content, and actors, which we applied as a starting point for our analysis. This is an example of a policy triangle model, which is frequently used to study different health issues in diverse geographical contexts (for a discussion of such models see [38, 39]). The four concepts making up the model could be understood as asking the questions why (context), what (content), how (process), and who (actors) regarding the policy under scrutiny [40]. Given that our analysis focused solely on the planning part of the policy process, we made certain adaptions to the original model and operationalized the concepts with specific types of events in mind, as described in Additional file 1. In Walt and Gilson’s [37] original framework, the concept of ‘actors’ focuses on actors important to the emergence and provision of a prescribed policy. In the aftermath of terrorism, however, we have to ask who the target populations of prescribed measures are. The content analysis was therefore expanded to include a thematic analysis employing both the initial four codes and inductive sub-categories developed during initial coding.

The analysis was conducted as follows: The first author performed the initial coding, including employing the deductive coding scheme and developing inductive sub-categories. To increase the internal validity of the analysis and counter researcher bias, the second author read the data material independently, before reading the first author’s analysis to check it for coherence and soundness. The analyses were then subject to repeated discussions, where the authors’ backgrounds in political science and medicine, respectively, were drawn upon to ensure an interdisciplinary approach.

## Results

Table 2 presents a comparative summary of the findings. We focused on measures that were relevant for terrorist attacks.

**Table 2:** Comparative summary

	Norway	France
Context	• Little focus on terrorism, more on disasters in general • References to plans and experiences from other countries	• Specific focus on terrorism, alongside other disasters • References to previous experiences in France, little from other countries
Process	• Local municipalities have much responsibility and flexibility to adapt healthcare responses • Responsibilities and organization in a crisis situation are based on the principles of responsibility, subsidiarity, and homogeneity • Immediate and long-term follow-up covered	• More centralized responsibility in larger geographical units, with pre-existing, disaster-specific networks regularly gathered and trained • Established mechanisms for powering-up the healthcare system in disasters • Strongest focus on the immediate to post-immediate aftermath
Actors - providers	• Several ministries involved, but no interministerial units • Multidisciplinary primary care based acute and long-term care	• Interministerial units established to respond to terrorist attacks • Specialized mental healthcare practitioners provide acute care and support
Actors - target population	• Main focus is on directly affected individuals. The term 'victim' is not used significantly • Groups in need of particular attention include children, youths, and minorities	• Main focus is on victims and their families, including the bereaved • Groups in need of particular attention include children
Content	• Disaster contingency is a continuation of the regular healthcare system: operations should be kept as normal as possible • Good planning, risk analysis, and training is central. Important tasks include the transmission of information to the public and involved actors • Basic care and practical help are more important in the emergency phase than therapeutic measures, with watchful waiting as a guiding principle. However, in the aftermath of the attacks a more proactive approach was selected	• Specific organization of the healthcare system in the emergency phase. Patients in need of continued care are to be directed into the regular healthcare system • Stronger focus on the organization of the healthcare system in the event of disaster than on the actual measures to be provided, but training of involved actors is central • Identifying and informing victims is stressed as important • Emergency care provided by specialized teams expected to follow state-of-the-art practices (although these practices are not necessarily specified)

### Context

#### Norway

The Norwegian material covered a wide array of disaster and crisis situations, and, apart from two documents published post-attack [41, 42], was not very terrorism specific. Pandemics and climate change were considered to be more likely threats to the Norwegian society than terrorist attacks [43]. The Norwegian approach to post-disaster psychosocial care was informed by the 2004 tsunami disaster in Southeast Asia which affected many Norwegian citizens [44, 45]. National plans and guidelines from Sweden, the UK, Australia, and the Netherlands were also utilized as a knowledge base. After the terrorist attack, knowledge on shootings and terrorist attacks, predominantly in the United States, was referenced [42].

Comprehensive knowledge of the disaster was considered important to organize help in the immediate aftermath [45]. It was stated that there will be individual variation in how disasters are experienced, and no time limit can be set on crisis reactions and grief [41]. It was specified that psychosocial follow-up should sometimes be considered healthcare, but not necessarily so [45]. This has implications for the legislation controlling follow-up. Finally, it was specified that services are required by law to be adequate, but that the extent, duration, and level of help required will need to be considered separately for each individual case [41].

The oldest document asserts that the lives of those who are affected by disasters will often change course [46]. Whereas the document from 2011 is more moderate, stating that crises and disasters are potentially traumatizing events, and that most people will be able to handle crisis situations without help [45].

#### France

The documents reflect that terrorism is assumed to be a persistent threat in France [47–49]. Significant attention is paid to how terrorist attacks are becoming increasingly complex, in terms of the weapons employed, and as multisite attacks. It is even suggested that, in order to increase resilience, the public could receive training in responding to terrorist threats.

Diverse events are mentioned as informing the French approach, including storms, floods, SARS, and heatwaves [50]. Furthermore, the 1995–1996 terrorist attacks in Paris have been important for the development of the current approach, as have experiences from subsequent terrorist attacks or conflict situations [48, 51]. Parts of the material reflect how terrorist attacks are considered to be war-like experiences, e.g. victims of terrorism may be entitled to a military invalidity pension applicable to civilian victims of war [47].

It is emphasized that beyond the physical injuries that can result from disasters, one may see psychological injuries requiring preventive emergency care, and that the subjective character of the trauma encounter and subsequent reactions must be considered [48, 51]. It is specified that terrorist attacks have a high potential for trauma due to their human intentionality, and that mental and physical injuries of being targeted with “weapons of war” are complex, which can complicate follow-up.

It is mentioned that the media coverage and other aspects of the socio-political context can affect how measures can be delivered after disasters [47, 49]. It is recognized that attacks are heterogeneous, and can occur in any location [48]. Furthermore, that disorganizing care structures can be a terrorist objective [49].

### Process

#### Norway

In Norway, disaster management within the healthcare system was largely organized within existing structures. The work was guided by three central principles: (1) responsibility: the institution that is normally responsible for a given service is responsible for contingency planning for this service, (2) subsidiarity: a crisis is to be handled at the lowest possible effective level, and (3) homogeneity: the organization that handles a crisis should be as similar as possible to the regular organization [44]. Given these principles, local government at the municipal level held central roles [43–46]. It was acknowledged that Norwegian municipalities are diverse, and that contingency planning needed to be tailor-made to the local context. The guidelines from 2008 claimed that increased complexity in society has led to new challenges for contingency work [43]. Municipalities were required to cooperate in crisis, but there was variation in whether this was controlled legally or based on recommendations from national authorities. Actually, a new nationwide principle of “samvirke” [52], implying collaboration and shared understanding, was promoted in the updated 2011 guideline on psychosocial care [45]. The 2011 update was published a month after the terrorist attacks, but was completed beforehand, and was thus central following the attack.

Time was recognized as a central challenge in disaster management. It was stressed that caring for those affected by disasters is a time-consuming task [46]. The documents covered typical reactions after disasters, including the differences between early and long-term needs. In the 2011 guidelines, watchful waiting was introduced as an important principle [45]. Here, it was also acknowledged that many of those affected by crises experience inadequate follow-up despite the mobilization of extensive resources. After the 2011 terrorist attacks, at least one year of follow-up was recommended [42]. It was stressed that the bereaved might take even longer to adapt. In the 2014 document, however, it was acknowledged that knowledge gathered internationally shows a need for follow-up for two to five years after a disaster [41]. Overall, the heterogeneity of needs was stressed.

#### France

In France, disaster and crisis contingency was mostly organized in plans and networks founded in the regular regional and zonal framework that could be activated during disasters. The networks were mobilized locally, but could also expand to regional or zonal levels, or seek reinforcements from neighboring departments (e.g. [49, 53, 54]). Exceptional health situations were therefore to be met with a graduated approach. Generally, these mechanisms were to be activated in exceptional situations only. Concurrently, there was an intention to create a continuum between the “normal” functioning of the health system and the operational response to large-scale crises.

The most important central planning mechanisms for organizing and controlling disaster and crisis contingency work were the ORSAN health plan and the ORSEC security plan. The ORSAN plan outlined how the health system should power-up during exceptional events that place the system under strain [53]. It formalized regional coordination of existing mechanisms in the health sectors, and coordination with other plans. It was specified that the number of victims are important measurements of an event’s gravity. The ORSEC plan is classified, and therefore could not be analyzed. Other documents described ORSEC as a relief organization program that organized the civil security response during disasters, including how to rescue large numbers of victims, manage a large number of casualties, and provide protection (e.g. [48, 49, 53]).

Additionally, public and private health facilities were legally obliged to have a White plan, to be activated when immediate mobilization was needed to handle a health emergency [49]. Furthermore, every department was required to have an Expanded White plan as a last resort, outlining all human and material resources that could be mobilized in a health crisis.

Although the French approach largely was based on central plans, it was acknowledged that local circumstances, available resources, and the events in question will vary [49, 53]. The ORSAN plan encouraged seeking the most effective approaches in any given territory. Similarly, the White plan specified that the Expanded White plans should be based on knowledge of risks and experiences specific to the relevant department.

Many of these mechanisms focused on the emergency phase and the need to act swiftly in the initial response. The long-term needs of those affected are not generally discussed at any length, as this is assumed to be the responsibility of the regular structures within the healthcare system which should adapt to meet individual needs [48, 51]. It was acknowledged, however, that reactions can be immediate or delayed.

### Actors

#### Norway

##### Providers

*Key coordinating actors *At the national level, the Ministry of Health had the overall responsibility for contingency planning and crisis management in the health and social services sectors, whereas its subordinate, the Directorate of Health, was responsible for ensuring that the different actors in these sectors cooperated in crisis and in contingency planning [43, 44, 55]. Additionally, the Ministry of Justice and the Police Directorate were important actors. The Ministry of Justice was the coordinating ministry after the terrorist attack, as is standard procedure during national crises in Norway.

County level government was responsible for giving advice and for coordinating contingency planning in cooperation with the municipalities [44]. Specialized healthcare remained the responsibility of the Regional Health Authorities (RHF) during disasters. Specialized healthcare was provided through agreements with the various health authorities, which consist of public hospitals and health institutions, or private actors.

Since crises should be handled at the lowest effective level of care, local governments at the municipal level played a central role as they are responsible for providing primary healthcare, including GPs and emergency primary care, to anyone within the municipality [43, 44]. Municipalities were thus required by law to have contingency plans.

*Key operational providers *The emergency medical communication centers organized under the RHFs could mobilize resources both within their own region and from other regions [44]. The police generally had the overarching responsibility for initiating rescue missions.

Municipalities would typically take part in rescue work and convene their crisis management team [44]. Most municipalities had a crisis team to provide psychosocial care to individuals, families, and local communities in crises/disasters [45]. Although the police were responsible for establishing reception and information centers, the psychosocial crisis teams could be central in operating them. After the 2011 terrorist attack, it was recommended that survivors from the attack on Utøya island should be assigned a contact person in their municipality to guide them into the healthcare system [56]. This person’s profession was decided locally. Occupational health services provided follow-up to survivors from the government quarters.

Operational healthcare can be broadly divided into primary healthcare provided by municipalities, and secondary healthcare, including hospitals and psychiatric outpatient clinics [43, 44]. Private actors could be involved if the municipalities were buying their services, but would not typically be central in crisis work [43]. Volunteer organizations could be mobilized, pending agreements with the affected municipality or at national levels. Religious actors, most prominently from the Norwegian church, have played important roles in psychosocial follow-up in Norway. Finally, there was a stated intention of facilitating military-civilian cooperation in times of crisis/disaster [55].

##### Target populations

In general documents, measures were intended for “the population”, without further specification [44]. More particular measures were intended for survivors or those directly affected, and their families including the bereaved. Others indirectly affected, e.g. through media, but also emergency personnel, volunteers, and journalists, were mentioned (e.g. [45, 46, 56]). The term “victim” was not used extensively. Groups identified as potentially in need of special attention were children and youths, including the children of those affected, minorities, refugees, and individuals with disabilities.

#### France

##### Providers

*Key coordinating actors *Key actors nationally were the Ministries of Health and its underlying Directorate-General for Hospitalization and the Organization of Care and Directorate-General for Health. The Ministry of the Interior, the Prime Minister’s Office, the Ministry for Foreign Affairs, and the Ministry of Justice were also central [47, 48]. In a disaster, the Prime Minister would activate an inter-Ministerial Crisis Cell, uniting all relevant ministries.

In the event of a terrorist attack, the Prime Minister could activate the Cross-Government Victim Support Unit, which included affected ministries. Additionally, victim support associations, the Guarantee Fund for Victims of Acts of Terrorism and Other Offenses, and a representative of the Paris Public Prosecutor could be represented. At the end of the crisis phase, the Minister of Justice could convene the inter-Ministerial Victim Follow-Up Committee, responsible for the organization of the post-crisis victim support system.

The regional health agencies (ARS) would organize the management of medical-psychological emergencies, including the region’s medical and psychological emergency units (CUMP) (e.g. [53, 54, 57]). The defense and security zones were central in the coordination of civilian and military efforts, responsible for non-military defense, and participated in crisis management when the means required to manage the situation exceeded the resources of the affected department. Additionally, regional zone agencies contributed to the mobilization of the national medical-psychological emergency network through mobilization of the CUMP in their zones.

Finally, the departmental prefects, the representatives of the national government in the departments, were responsible for public order and the protection of populations [47]. They were Directors of Emergency Operations and oversaw all relief and policing missions.

*Key operational providers *The key operational actors in the emergency phase were the fire and rescue services together with the police and military police. The French fire brigade has an integrated medical rescue service. There are also fire brigade personnel who are trained to intervene in securitized zones, including medical teams providing early treatment in zones where regular rescue teams cannot enter due to safety restrictions [48].

Pre-hospital care was the responsibility of the Urgent Medical Aid Service (SAMUs), and their mobile emergency and resuscitation teams [53]. Each health facility with a SAMU had a CUMP composed of trained mental health professionals, such as psychiatrists, psychologists, and psychiatric nurses [51, 54, 57]. Their role was to ensure that victims received medical-psychological care in the immediate and post-immediate phase after disasters. In large scale events, the national network of CUMPs could be mobilized.

External resources, including army medical centers, the French Defense Health Service, and army teaching hospitals could also be involved [53]. Private actors would not usually be directly involved in disaster work, but could be involved indirectly through relieving involved healthcare workers.

Finally, other departmental victim support services, such as victim support associations approved by the Ministry of Justice and approved civil security associations could cooperate with the CUMPs through formalized partnerships [51].

##### Target populations

Protection of the population was a central aim, including how a population may be indirectly affected by large-scale events. The more specific measures were directed towards victims and their relatives, including bereaved families [47]. It was specified that victims can be both physically and psychologically injured.

Affected children, including children of deceased or injured victims who are unable to care for them, were mentioned specifically. Specific attention was given to families of deceased victims living abroad, to French nationals victimized abroad, to witnesses to the events, and to health professionals and rescue personnel.

The term victim was used in several documents, defined in accordance with the 1985 Declaration of the United Nations of Basic Principles of Justice for Victims of Crime [49]. Compiling a list of victims of terrorism received significant attention, and was considered a vital step in reaching victims and ensuring that the right individuals received the compensation and assistance they were entitled to [47].

### Content

#### Norway

A central point of departure for the Norwegian measures was that operations should be kept as normal as possible, even in crisis [43, 44]. Concurrently, it is specified that crisis organizations should be established rapidly. Under some circumstances this would involve reorganizing or expanding regular operations.

There was a focus on the need for good planning, including risk analysis which accounts for local circumstances and suggestions regarding the contents of training for those involved [43, 44]. It was stressed that providing care to those affected by disasters is a comprehensive task. The oldest document specified that post-disaster care involves psychological, physical, social, spiritual, and material dimensions [46]. Coherence between different actors was highlighted as important, as was their ability to cooperate with efficiency. There were specifications regarding the requirement for contingency planning, and what these plans should include, but not very specific descriptions of how the municipalities should organize this. The municipalities held a wide range of responsibilities in a crisis, ranging from maintaining health and social services, to reporting about the situation to other levels of government [43, 45]. Simultaneously, they were required to continuously assess whether reinforcements from other municipalities, or other levels of government, were needed.

Information was reiterated as important, including how information should be distributed to relevant actors at all levels and the necessity of providing those affected and the general public with reliable and sufficient information [43, 45]. Interaction with the media, both by authorities and affected individuals, was discussed.

The municipalities held much responsibility for establishing teams for psychosocial follow-up and deciding how they should operate. Still, the documents were quite detailed on the measures to be provided immediately after a disaster, including the definition of psychosocial measures and how they should be provided by psychosocial crisis teams and others. The guideline for psychosocial follow-up published in 2011, underlined that there are few studies on the effects of early post-disaster interventions, and that these are often based on expert opinions [45]. These guidelines stressed the importance of taking evidence-based knowledge into account.

Information, basic care, and practical help were highlighted as particularly important in the immediate aftermath to meet the needs of affected individuals and facilitate natural healing [45]. The principle of watchful waiting was central. Therapeutic interventions were recommended only in exceptional circumstances. It was pointed out that whereas physical injuries should be treated immediately, psychological screening was not necessary in the immediate aftermath, and that the psychosocial crisis teams should be careful not to replace regular social networks. Concerning physical injuries, criteria were set out at each hospital for when and how they should declare a disaster warning, which could be associated with a call-back of personnel and cooperation with other hospitals.

It was reiterated that help should be customized to the individual, and that there are no guidelines for a general follow-up of everyone affected by the same disaster. However, the municipalities were recommended to proactively contact everyone directly affected by the terrorist attack on Utøya island to get an overview of individual needs, provide information about how to seek help, and offer a standardized screening at least three times in the year following the attack [56]. A designated contact person in the municipality was to initiate this contact. This model was developed specifically for the aftermath of the 2011 attack, and it was specified that this recommendation could be adjusted to local circumstances in the municipalities.

For long-term follow-up, specific therapeutic approaches were recommended. In the general guideline, it was suggested that psychosocial follow-up should be integrated into the regular healthcare system as much as possible [45]. Nonetheless, about three years after the terrorist attacks, the need for long-term follow-up was stressed [41].

#### France

The central components of the French approach were provision of emergency services and medical-psychological care in the first days and weeks after a disaster [53]. There was a stated aim of directing victims to the regular healthcare services when the emergency phase was over [51].

The plans were more focused on the organization of healthcare than its content. They described how the healthcare actors could power-up in case of unusual strain, how they could coordinate with other plans, which resources were available, and how they could be mobilized [49, 53, 54]. The ORSAN plan outlined how the healthcare system should provide care in exceptional situations through the private sector, pre-hospital phase, and in healthcare facilities [53]. There was extensive focus on guidelines for training healthcare professionals, sometimes partly organized nationally.

It was stressed that several health, judicial, and administrative measures must be combined to meet the needs of those affected by terrorism [47, 48]. It was outlined how the involved actors should react, including the responsibility of the departmental prefect for ensuring the immediate mobilization of emergency relief, medical assistance, and security at the site of the attack. Furthermore, it was specified how the medical and psychological care of victims should take place in parallel. The provision of information to victims and their families was highlighted as important. Victims of terrorist attacks were exempt from all health costs directly related to the attacks. Consequently, documentation was important, as were procedures for identifying victims and classifying acts as terrorism.

The CUMPs were responsible for medical-social care in the first days to four weeks, and, if necessary, guiding patients into longer term care [51, 54, 57]. Care should be provided according to best practices. What constituted best practice was not specified. The CUMPs were responsible for issuing a certificate of medical and psychological injury and providing information about expected reactions and how to contact the healthcare system.

## Discussion

What emergency preparedness in the public health sector should look like is debated [13, 14]. There is no accepted framework for how national health systems should prepare for handling the human consequences of terrorist attacks. Still, there is current political interest in increased cross-border cooperation in addressing health threats in Europe [5]. The findings of the current paper sheds light on how national contexts represent both possibilities and potential pitfalls for such coordination. We have analyzed how two generally peaceful democracies in Europe prepared for the health consequences of terrorist attacks. Although we found several similarities, there were also important differences. Given the existence of international guidelines we could have expected the content of the prescribed measures to be somewhat similar. At the same time, previous studies have found that there have been differences between countries in Europe when it comes to their psychosocial care responses after terrorism [20]. Following this, and due to differences in the political and healthcare systems, we anticipated that the context in which the approach to disaster follow-up had developed and existed, and the actors responsible for providing the prescribed measures, would vary.

Emergency planning is influenced by challenges and opportunities unique to each political system (Perry and Hirose, 1991, as referenced in [6]). This paper provides several examples of how the characteristics of political and healthcare systems may influence approaches to follow-up. This includes, the sheer size of the population, the size of the administrative units responsible for providing services, and, subsequently, how the systems are organized in both non-disaster and disaster contexts. The French system is more centralized, with prefects who represent the central government at the sub-national level (for a discussion of the prefects role see [24]). This is reflected in the documents through rather detailed descriptions of how relevant actors should be organized in case of disaster, and how responsibility can be raised to a higher level of administration when necessary. In the Norwegian documents, there are arguably signs of stronger local autonomy in disasters. This reflects the general organization of the political system in Norway [24]. Norway’s geographically dispersed population may partly explain why local autonomy appears to be the preferred approach in much contingency work.

Although local autonomy appears to be stronger in Norway, the Norwegian documents describe more detailed the specific measures to be implemented and their associated knowledge base. One interesting comparative aspect in this regard, is the difference in how the units responsible for providing psychosocial follow-up in the acute phase are described in the two countries. Whereas the Norwegian guideline for psychosocial follow-up provides detailed information on what the crisis teams should do in disasters, the CUMP documents on the French side are more specific in their presentation of the professions than their duties. Their intended role appears to be more open to the discretion of the CUMPs themselves, which are organized in a national network that might facilitate exchange of experiences and discussion of best practices. This means that Norway has prioritized having services that are close to where the receivers live, while France has prioritized services with specialized mental health competence.

This difference is linked to the important question of which challenges the authorities identify as priorities to address with a prescribed policy. As mentioned in the introduction, policies are intended to solve specific challenges within a certain amount of time [12]. Protecting the health and psychosocial wellbeing of those affected are typically identified as important tasks for the healthcare system after terrorism [4, 7, 16]. This is reflected in both countries’ documents, however, their approach differs. Defying expectations based on the existence of international guidelines [18, 19], the content of the prescribed measures varied in the two countries. As alluded to above, one of the most distinct differences is the degree to which specialized mental healthcare is relied upon to provide psychosocial follow-up in the emergency phase. In the Norwegian guidelines, it is stated that only exceptional cases will require psycho-therapeutic treatment in the first week after a potentially traumatic event. This is part of the rationale behind why the psychosocial crisis teams are composed of an interdisciplinary group trained to provide care in the immediate aftermath, but usually not, for instance, psychiatrists prepared to provide treatment. In France, on the other hand, the CUMPs consist of psychiatrists, psychologists, and psychiatric nurses specifically, in other words, professionals who specialize in providing psychiatric treatment and care. Different approaches to provision of health services can potentially be explained by political or systemic factors, or even values [58] or pre-existing expectations. The Inter-Agency Standing Committee (IASC) Reference Group for Mental Health and Psychosocial Support in Emergency Settings [17], have suggested a framework for mental health and psychosocial support in emergency situations, which includes six core principles (p. 5). While both countries’ plans and guidelines reflect these principles, including for instance building emergency preparedness on available resources and capacities, the countries diverge from each other when it comes to the extent to which they adhere to the sixth principle, which is “multilayered support”. This involves an acknowledgement of the heterogeneous needs that will be present across an affected population following a disaster, which will lead to a need for differentiated support responses. These could, according to IASC, be organized according to an intervention pyramid, which has basic services and security at its foundation and specialized services at the top [17]. Arguably, however, this pyramid appears to have a stronger presence in the Norwegian than the French approach.

Beyond their political and healthcare systems, the countries’ national histories of terrorism could be important to understand their approach to post-terror follow-up. Experiencing such adverse events might alter the space for political maneuvering, and this may in turn be important in order to understand subsequent policies and planning. Kingdon [2] has described how particular issues typically emerge on political agendas due to being identified as problems e.g. through what he calls a “focusing event”, meaning a crisis or a similar symbolic event (pp. 94–98). Following Kingdon [2], Solheim [59] also asserts that terrorism may change public attitudes, thereby introducing terrorism into what Kingdon [2] calls the politics stream, where room is created for policies to emerge. The third stream introduced by Kingdon [2], the policy stream, is arguably also relevant for follow-up in the healthcare system after terrorism, as knowledge on the consequences of terrorist attacks, and the associated follow-up, continuously accumulates through research and national or international experiences. Responses to terrorist attacks provide opportunities for learning [4]. Hence, plans for meeting healthcare needs after terror may develop in the intersection between the best practice for addressing specific needs as identified in international guidelines, research literature, and previous experiences, and the national political framework. Terrorist attacks are more present in the French documents as potential threats, requiring specific preparation, than in the Norwegian documents published prior to the attacks there. Kingdon’s [2] framework is useful for understanding why. It is reasonable to assume that this reflects more experience in France with terrorism, which in turn pushed these preparations into policy agendas to a greater extent than in Norway prior to 2011.

It is important to remember that planned measures are not necessarily implemented, and that policy plans are not accurate representations of what actually occurs during crisis management. As pointed out by Perry and Lindell [6], the written documents are only one part of the process, and emergency plans should not automatically be understood as emergency preparedness. Informal processes not visible in written documents may also be a significant part of emergency preparedness. Furthermore, plans are only acted upon if their content is known to relevant actors. Previous research has found that this knowledge can be limited [60]. Still, planned measures are important to understand the intentions and reasoning behind actions, as well as the priorities on which they are based. To the extent that plans are publicly known, they may also set a precedent for a population’s expectations.

### Strengths and limitations

Systems for follow-up after terrorist attacks are complex, involving different phases and many actors. In this article, we have covered key features of these systems in Norway and France. Although this provides a comparative overview, it does not capture every process involved in crisis management after terrorism. The current study focused on policies and plans at the national level. There will also be relevant plans at sub-national levels, which could not be accounted for in this study. Nevertheless, it can be argued that, in the case of disaster preparedness, it is relevant to study what is prioritized and controlled at the national level of government.

## Conclusion

This article demonstrates that there is probably no single approach to contingency planning for healthcare systems in the case of terrorist attacks that will be applicable in all contexts. Historical and systemic factors influenced the extent to which the specific response to terrorist attacks was planned beforehand, how it was done, and by whom. The international knowledge base of good practices to meet needs following terrorist attacks is only one of several factors influencing how countries approach this challenge. Our findings leave us questioning the actual application of common international guidelines for contingency planning in the healthcare system. Notwithstanding, core elements of good practice can be implemented internationally. An important first step could thus be to map out existing services and practices across countries to identify and formulate potential core elements for follow-up in the acute and long-term aftermath of terrorism. Similarly to how terrorist attacks are important for understanding subsequent disaster response in the countries in which they occur, they also represent important opportunities for learning in an international context.

## Supplementary Information

**Additional file 1.** The operationalization of the concepts in the health policy model for the current analysis.

## Data Availability

All data material analyzed in this article is publicly available and is referenced in the results section and included in the reference list.

## Abbreviations

ARS: Regional health agencies (France)
CUMP: Medical and psychological emergency units (France)
GDP: Gross domestic product
GP: General practitioner
IASC: Inter-Agency Standing Committee Reference Group for Mental Health and Psychosocial Support in Emergency Settings
RHF: Regional Health Authorities (Norway)
SAMU: Urgent Medical Aid Service (France)
