# Supplementary material for: Preparing for the unexpected: a comparative study of policies addressing post-terror health reactions in Norway and France
Source: Int J Ment Health Syst. 2023 May 26;17:13. doi: 10.1186/s13033-023-00582-x (PMC10214657; doi:10.1186/s13033-023-00582-x)
Supplement: Supplementary file 1 — Additional file 1. The operationalization of the concepts in the health policy model for the current analysis. [file 13033_2023_582_MOESM1_ESM.docx]

**Additional file 1: The operationalization of the concepts in the health policy model made for the current analysis**

|  | ***Description of code*** | ***Inductive categories*** |
| --- | --- | --- |
| **Context** | - References to previously identified needs after terrorist attacks. - Relevant background factors in the political/historical/healthcare systems |  |
|  |  |  |
| **Process** | - Characteristics of national health care systems - Organization and harmonization of disaster specific measures with and within the regular system. - Timing of measures suggested - Different phases in the aftermath and their coverage by (different) plans, and/or acknowledgment in plans. |  |
|  |  |  |
| **Content** | - Type of actions/measures prescribed. - Specific content, information to be included in prescribed plans. - The knowledge base of measures prescribed. |  |
|  |  |  |
| **Actors** | - Involved actors | - Providers: Coordinating and operational - Target populations |
